# Relationship among ICD-11 personality pathology and related concepts with somatic symptoms among Croatian psychiatric patients

**DOI:** 10.1192/j.eurpsy.2026.11466

**Published:** 2026-07-31

**Authors:** N. Jakšić, I. Simunovic Filipcic, M. Rimac, M. Grah, I. Filipčić, D. Marčinko

**Affiliations:** 1 Department of psychiatry and psychological medicine, University Hospital Centre Zagreb, Zagreb; 2 Faculty of Dental Medicine and Health, Josip Juraj Strossmayer University of Osijek, Osijek; 3 School of Medicine, University of Zagreb; 4 University Psychiatric Clinic Sveti Ivan; 5 University of Applied Health Science, Zagreb, Croatia

## Abstract

**Introduction:**

Somatic symptoms are often highly prevalent and intertwined with personality pathology in patients suffering from psychiatric disorders.

**Objectives:**

This study aimed to investigate the associations between ICD-11 personality disorder severity, maladaptive trait domains, hypomentalization and childhood trauma with the intensity of somatic symptoms.

**Methods:**

The sample included 469 individuals (M age = 44.39; 61% females) with psychiatric disorders, including personality disorders. Participants completed self-report measures assessing ICD-11 personality disorder severity, ICD-11 personality domains, reflective functioning/mentalization and various forms of childhood traumatic experiences. All the analyses controlled for age, sex, education level and somatic comorbidities.

**Results:**

Personality disorder severity, trait negative affectivity, hypomentalization, and childhood emotional abuse were each significantly associated with somatic symptoms. Mediation analyses revealed that personality disorder severity, negative affectivity, and hypomentalization partially mediated the relationship between childhood emotional abuse and somatic symptoms. In the final parallel mediation model, only personality disorder severity and negative affectivity remained significant mediators, while the effect of hypomentalization was no longer significant.

**Conclusions:**

These findings indicate that personality disorder severity and negative affectivity play a central role in linking childhood trauma with the manifestation of somatic symptoms. This highlights the importance of assessing and targeting core personality pathology and affective dysregulation in patients presenting with prominent somatic complaints.

**Disclosure of Interest:**

None Declared

